# Extent, impact, and predictors of diagnostic delay in Pompe disease: A combined survey approach to unveil the diagnostic odyssey

**DOI:** 10.1002/jmd2.12062

**Published:** 2019-07-17

**Authors:** Florian B. Lagler, Angelika Moder, Marianne Rohrbach, Julia Hennermann, Eugen Mengel, Seyfullah Gökce, Thomas Hundsberger, Kai M. Rösler, Nesrin Karabul, Martina Huemer

**Affiliations:** ^1^ Institute for Inborn Errors of Metabolism, Paracelsus Medical University Salzburg Austria; ^2^ Department of Paediatrics Paracelsus Medical University Salzburg Austria; ^3^ Division of Metabolism and Children's Research Centre University Children's Hospital Zurich Switzerland; ^4^ Division of Metabolic Diseases (Villa Metabolica) Center for Diseases in Childhood and Adolescence, Mainz Medical University Mainz Germany; ^5^ Department of Neurology Cantonal Hospital St. Gallen Switzerland; ^6^ Department of Neurology, Inselspital University Hospital Bern Switzerland; ^7^ Center of Endocrinology and Metabolism, Rheumatology and Neurology Endokrinologikum Frankfurt Frankfurt a. M. Germany; ^8^ Radiz – Rare Disease Initiative Zürich, Clinical Research Priority Program University of Zürich Zürich Switzerland; ^9^ Department of Paediatrics Landeskrankenhaus Bregenz Austria

**Keywords:** Pompe disease, diagnostic odyssey, diagnostic delay, expert centers, initial symptoms, patient perspective, survey

## Abstract

**Background:**

Early diagnosis is of substantial benefit for patients with Pompe disease. Yet underdiagnosing and substantial diagnostic delay are still frequent and the determinants of this are unknown. This study is the first to systematically investigate the diagnostic odyssey in Pompe disease from patients', parents', and physicians' perspectives.

**Methods:**

Patients with infantile or late onset Pompe disease, their parents as well as their metabolic experts were invited to fill in respective surveys. The survey addressed perceived disease symptoms at onset and during the course of the disease, specialties of involved physicians, activities of patient‐initiated search for diagnosis and the perceived impact of time to diagnosis on outcome. Results of experts' and patients'/parents' surveys were compared and expressed by descriptive statistics.

**Results and Discussion:**

We collected data on 15 males and 17 females including 9 infantile and 23 late onset Pompe patients. All received the correct diagnosis at a metabolic or musculoskeletal expert center. Patients with direct referral to the expert center had the lowest diagnostic delay, while patients who were seen by several physicians, received the correct diagnosis after 44%‐200% longer delay. The proportion of direct referral varied strongly between pediatricians (57%) and other disciplines (18%‐36%).

**Conclusion:**

Our study highlights a substantially larger diagnostic delay in Pompe patients that are not directly referred to expert centers for diagnostic work. Our findings may be used to develop more successful strategies for early diagnosis.

**Synopsis:**

Diagnostic delay in Pompe disease is substantial particularly in patients that are not directly referred to expert centers for diagnostic workup, so facilitating direct referral may be a new strategy for early diagnosis.

**Abbreviations**
ERT: enzyme replacement therapy
GAA: acid alpha glucosidase
HCP: health care provider
IOPD: infantile onset Pompe disease
LOPD: late onset Pompe disease
OMIM: online mendelian inheritance in man

## 1 INTRODUCTION

Pompe disease (glycogen storage disease type II; OMIM #232300) is a progressive, debilitating, and potentially fatal myopathy. It is caused by an autosomal recessive inherited deficiency of the lysosomal enzyme acid alpha glucosidase (GAA) and is estimated to affect 1:40 000 individuals.^1^ GAA deficiency leads to intralysosomale and intracellular accumulation of glycogen, alteration of autophagy and cell signaling, and consecutively to progressive skeletal and cardiac muscle cell dysfunction and death.^2^, ^3^, ^4^, ^5^ The phenotypic spectrum is broad and ranges from classic infantile Pompe disease (IOPD) to attenuated, late onset forms (LOPD). In IOPD, glycogen storage starts even before birth and most affected children become symptomatic within the first weeks of life. If untreated, IOPD progresses rapidly to fatal cardiac and respiratory failure mostly within the first two years of life.^5^ The incidences of IOPD and LOPD have been estimated to 1 in 138 000 and 1 in 57 000 births, respectively.^1^, ^6^, ^7^ LOPD may present any time from childhood to old age with initially nonspecific symptoms. The course of LOPD is highly variable. Delayed achievements of motor milestones, recurrent severe respiratory infections, failure to thrive/weight loss as well as impaired exercise tolerance, fatigue, and signs of progressive myopathy and respiratory insufficiency have been observed. Cardiomyopathy is not a characteristic feature of LOPD.

At present, Pompe disease is treatable but not curable. Enzyme replacement therapy (ERT) has a significant positive effect on ventilator free survival time and heart muscle in IOPD patients and may alter the clinical course favorably in LOPD. Early diagnosis seems to be of significant value.^8^, ^9^ Additionally, setting the diagnosis of Pompe disease improves quality of care by opening the gate to specialized multidisciplinary centers and genetic counseling services.^10^, ^11^, ^12^ Unfortunately, poor recognition, underdiagnosing,^13^ and substantial diagnostic delay are still frequent and a number of awareness raising campaigns showed no obvious effects.^11^, ^12^, ^14^ Yet determinants of diagnostic failure or delay have not yet been systematically studied. This motivated us to explore patients', parents', and physicians' perspectives on factors associated with diagnostic delay as a first step toward improved strategies to reduce the diagnostic delay in Pompe disease.

## 2 METHODS

### 2.1 Participants

Experts in pediatric and adult metabolic medicine following patients with Pompe disease from six centers in Austria (Bregenz, Salzburg), Germany (Mainz), and Switzerland (Bern, St. Gallen, Zurich) agreed to participate and recruit patients/parents for the study. The participating sites included a very large center as well as medium and small‐size centers. After the respective institutional review boards (Salzburg, Vorarlberg, Zurich, Rhineland Palatinate) had given their approval (415‐EP/73/332‐2014; EK‐Nr. 2014‐2/2; KEK‐ZH‐Nr. 2014‐0111, 837.277.14[9516]), all patients with proven Pompe disease and their parents were invited to participate. All participants gave informed consent and completed a specifically developed survey.

### 2.2 Data collection

Specific surveys for patients, parents, and experts (as a case report form for chart review) were made available for online data entry via Survey Monkey (https://de.surveymonkey.com/). To minimize selection bias, a printed copy of the survey was provided to patients/parents who preferred this to the online tool.

### 2.3 Data analysis

Printed copy data were entered into the database by the study team. Data were analyzed using the SPSS version 21 software. Categorical data were expressed as proportion of total number of patients in the respective disease form group. Continuous data were calculated as mean, median, minimum, and maximum. Correlations were calculated after rank‐transformation using the Spearman‐rank method. *P*‐values less than .05 were considered significant.

### 2.4 Definitions

Patients with onset of symptoms during the first year of life were classified as IOPD patients; the remaining patients were classified as LOPD. Diagnostic delay was defined as the time between onset of symptoms and setting of the diagnosis. The term “diagnostic odyssey” in Pompe disease indicates a patients' journey from the first contact with a health care provider (HCP) to the expert center that are defined as metabolic (infants or children) or neuromuscular centers (adults).

## 3 RESULTS

We collected data on 15 male and 17 female patients. Nine patients were classified as IOPD, 23 as LOPD. Twenty‐one patients and 11 parents (2 fathers, 8 mothers, 1 missing information) completed the respective surveys. Seven experts commented on five IOPD and 22 LOPD cases.

### 3.1 Involved HCP

Patients with IOPD had been seen by general pediatricians (67%), physical therapists (22%), and neurologists (11%) before referral to a metabolic or neuromuscular expert. LOPD patients had consulted general practitioners (50%), neurologists (40%), physical therapists (20%), orthopedists (15%), and pediatricians (5%). Patients with IOPD consulted 1‐2 (median 1) different HCP before referral to the metabolic center as compared to 1‐6 (median 2) HCP in LOPD.

### 3.2 Diagnostic delay

In IOPD patients, parents retrospectively dated disease onset in their child to the age of 7 months in median (range 0‐12 months). The correct diagnosis was set with a delay of 2.5 (1.5‐6) months.

Twenty‐two percent of patients/parents with a diagnostic delay between 6‐12 months (median 6 months) considered this diagnostic delay inacceptable and associated with negative consequences on disease outcome. Sixty‐seven percent of IOPD patients/parents judged the delay as acceptable or satisfying.

**Figure 1:**
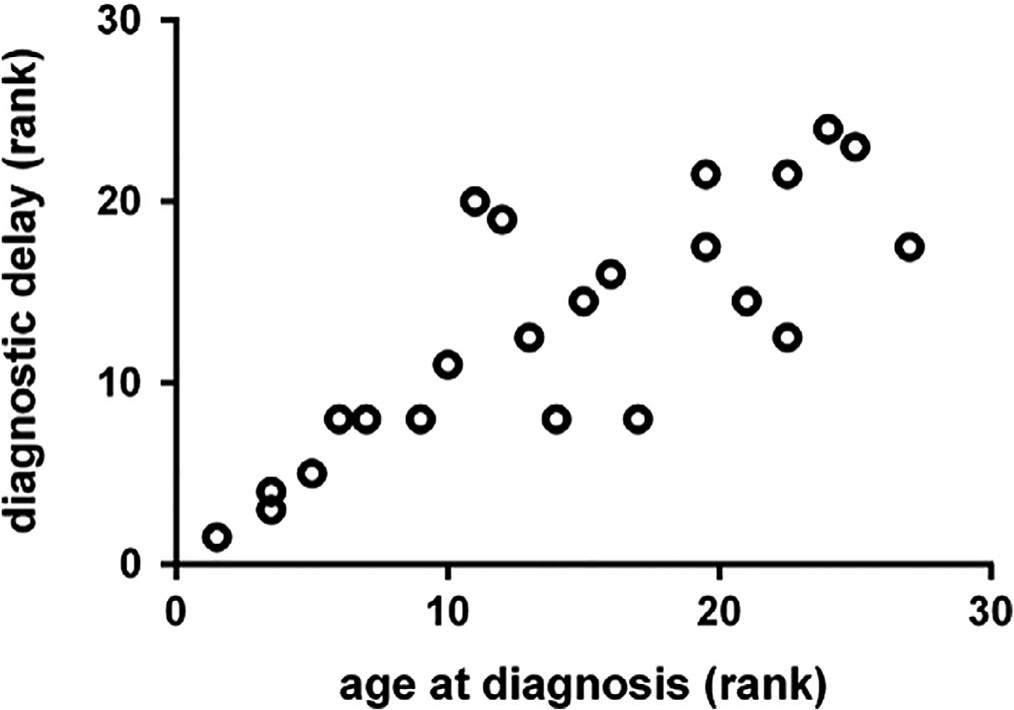
Rank correlation (Spearman) of diagnostic delay and age of onset

LOPD patients were diagnosed 144 (12‐480) months after the onset of first symptoms at a median age of 26 (3‐60) years. The delay was acceptable for 22% and satisfying for 39% of patients/parents. One (4%) participant considered the delay inacceptable but provided no information on its extent. Forty‐three percent of LOPD patients/parents stated that the diagnostic delay had no negative consequences on outcome while 17% felt the long diagnostic delay was “crucial” for their current physical status. Figure 1 illustrates that age at onset and diagnostic delay are positively correlated (*R* = .82).

Twelve of the complete sample of 32 patients had been referred directly to an expert center after initial assessment by a pediatrician (4), general practitioner (4), neurologist (2), orthopedist (1), or internist (1).

**Figure 2:**
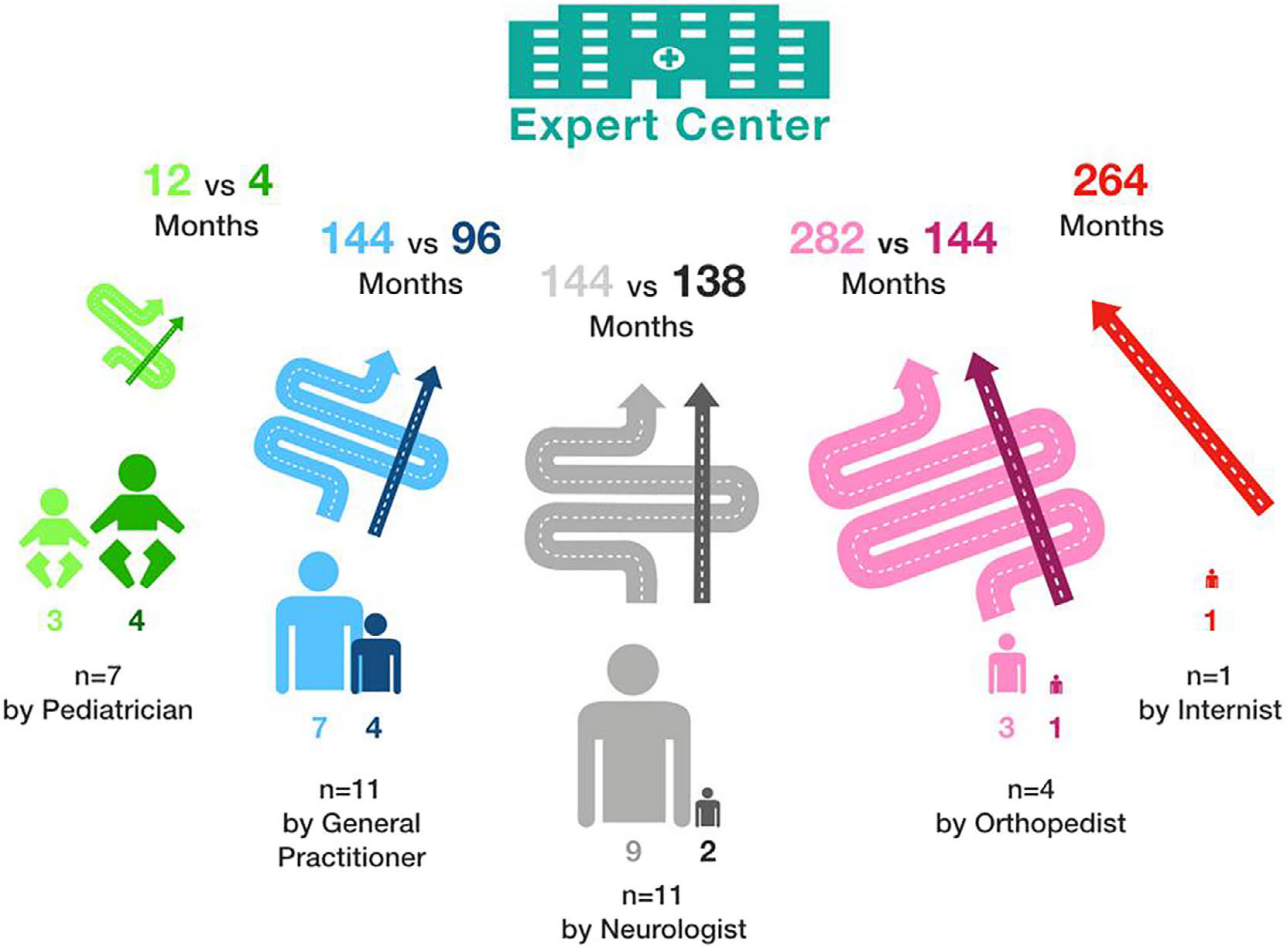
Diagnostic delay by HCP involved. The figure shows which specialists (lower part) have been consulted for the initial signs and symptoms of Pompe disease, the number of patients directly referred to an expert center (straight arrows in dark colors), and the number of patients who underwent a diagnostic odyssey (winded arrows in light colors). In the top part the median diagnostic delay in months is compared between the latter two groups indicating approximately a duplication of delay by the diagnostic odyssey

Twenty of 32 patients had been seen by 2 to 6 different HCP before referral to the expert and diagnosis. Overall diagnostic delay was less in IOPD and LOPD patients referred directly to the expert center (1.5‐264, median 112 months) as compared to other referral strategies (1.5‐480, median 178 months). Serial referral to nonexpert HCP increases the median diagnostic delay in IOPD and LOPD patients primarily seen by pediatricians from 4 to 12 (+200%) months and in all other patients from 132 to 190 (+44%) months in median. Figure 2 illustrates the delay to diagnosis in relation to the diagnostic odyssey and highlights substantial differences between patients initially seen by pediatricians and those seen by other HCP.

### 3.3 Symptoms at onset and during the disease course

**Figure 3:**
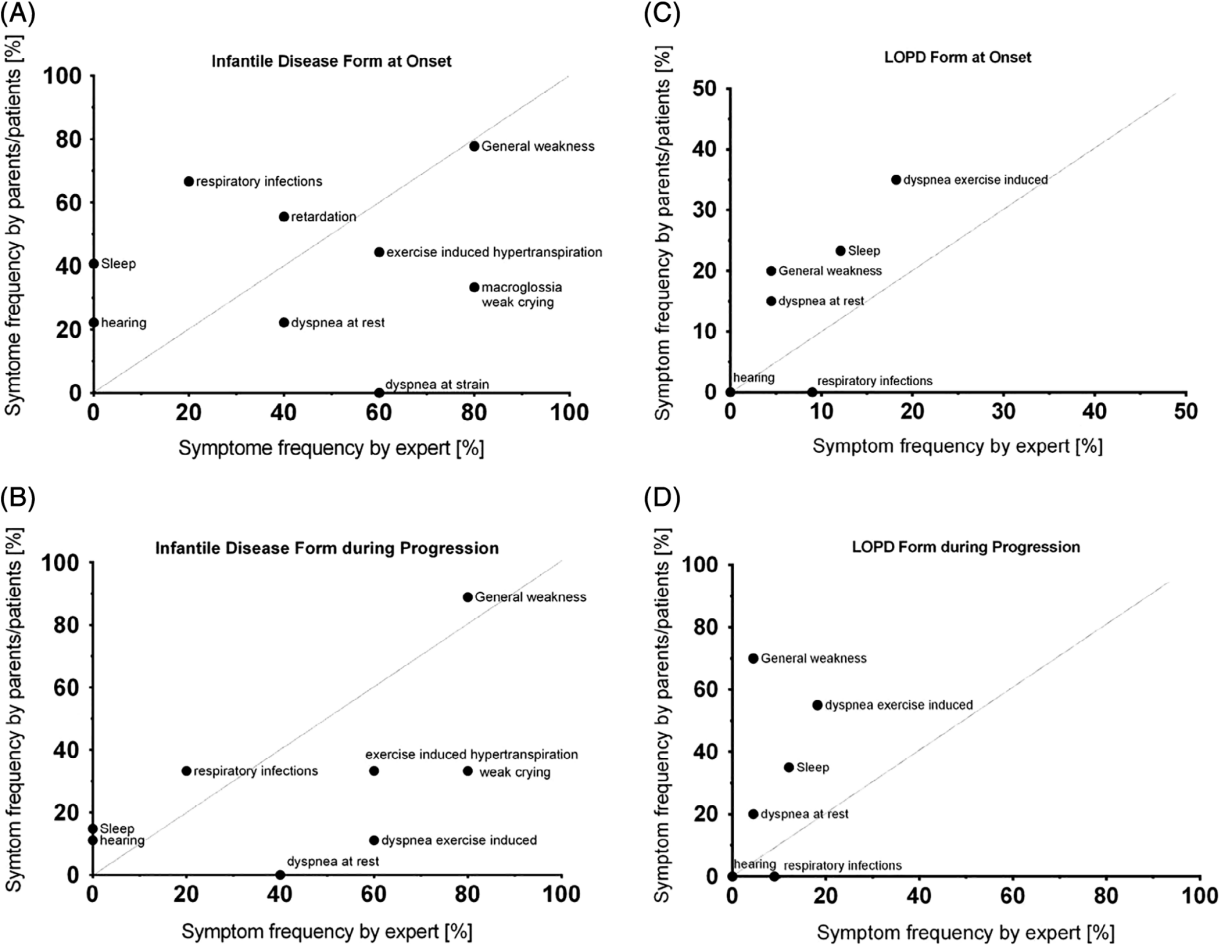
Symptoms as specified by parents/patients vs experts

Expert chart review revealed generalized muscular hypotonia, weak sucking and crying, hypomimic face and macroglossia as leading initial symptoms in 80% of IOPD patients (Figure 3A). During the course of the disease additionally motor retardation, activity‐induced tachypnoea/dyspnoea, excessive sweating as well as respiratory infections were noticed in 80% of IOPD patients.

In LOPD, experts perceived muscle weakness of hips/legs and trunk (86 and 77%, respectively), reduced performance in sports (73%), and difficulties to cope with every day‐life (68%) (Figure 3B) as most common first symptoms of LOPD. During the course of the disease, main limitations and symptoms were diminished resilience in school/job (86%), back pain (64%) sleeping disturbances and nonrestorative sleep (59% and 55%), fatigue (59%), weakness of the arms (59%), and exercise‐induced tachy−/dyspnoea (63.6%).

Interestingly, symptom patterns differed only slightly between patients' and experts' reports (Figure 3C,D). In IOPD, experts reported weak crying and signs of dysphagia much more frequently than parents did. In LOPD, patients/parents reported drooling more frequently than experts did. Otherwise, leading symptoms were quite congruent.

As expected, the prevalence of symptoms increased during the course of disease underlining the progressive character of the disease.

### 3.4 Inaccurate diagnoses

Eleven of 32 (34%) of the patients reported on incorrect diagnoses they had received before seeing the metabolic or neuromuscular expert. In LOPD “unclear muscle dystrophy/hypotonia/weakness” was reported most frequently (5/11) followed by “ankylosing spondylitis/degenerative back disease” (2/11). “Depression”, “fatigue,” and “gait abnormalities” were considered in individual patients. In IOPD the diagnoses “unclear weight loss,” “mononucleosis,” “birth injury,” and “hip dysplasia” were reported.

### 3.5 Self‐initiated search for diagnosis

Three parents of IOPD patients and six LOPD patients (36%) had performed a self‐initiated internet search. The predominantly used search‐engine was Google, followed by Yahoo. A single patient referred to Pubmed as primary source for information. One LOPD patient consulted medical literature, and one patient contacted medical institutions for help.

## 4 DISCUSSION

This study is the first approach to systematically investigate the diagnostic odyssey in IOPD and LOPD, its association with diagnostic delay and perceived impact on outcome from a patients' / parents' as well as the expert perspective. Our results indicate that a diagnostic odyssey with involvement of several HCPs is associated with a substantial increase of diagnostic delay (+ 44%‐200%). This encourages the development of novel strategies to improve the timely diagnosis of Pompe disease and other rare diseases.

Our data are in good accordance with previous, partly very large studies [14] with regard to the following findings: IOPD with cardiomyopathy was the smallest patient fraction (11%) in our sample with the earliest onset (1.5‐3, median 3 months), a rather uniform clinical pattern and a rapidly progressive course. Diagnostic delay (1.5‐3, median 2.5 months) was shortest in this group, probably owing to the course characteristics and the resulting diagnostic and therapeutic impact.

In contrast, LOPD represented 67% and probably due to the heterogeneous presentation and course—showed a substantial diagnostic delay (12‐480, median 144 months). Additionally, symptoms at presentation and throughout the disease course as collected by expert chart review and the rate of misdiagnosing (34%) were congruent with published data (http://www.eurordis.org/IMG/pdf/voice_12000_patients/EURORDISCARE_FULLBOOKr.pdf) indicating sufficient representativeness of our sample.

Patients' views have rarely been studied^9^, ^15^, ^16^, ^17^ and thus our results may offer options for new awareness raising strategies for Pompe disease.

Our hypothesis that patients'/parents' views and description of first symptoms might differ substantially from textbook knowledge on Pompe disease and may thus be misleading for physicians in the first place was not supported by our data. The description of initial symptoms differed only slightly between patients/parents and physicians and no novel red flag symptoms/terms of description could be identified.

Self‐initiated search for diagnosis was surprisingly infrequent in our study. However, most participating patients had been diagnosed before the internet became easily accessible and widely used. Like in other rare conditions^18^, ^19^ provision of information via the internet and social media may be helpful in directing Pompe disease patients to the expert.

Beyond pediatricians and neurologists, Pompe disease patients in our study first encountered also physical therapists, general practitioners, and orthopedists. These professions have so far not been and could in the future be taken into account as target groups for awareness‐raising campaigns in Pompe disease.^20^

All patients finally received their correct diagnosis in a center with expertise in metabolic or neuromuscular diseases. The proportion of patients directly referred to an expert center varied substantially, dependent on the HCP seen first (18% direct referral by neurologists to 57% by pediatricians) (Figure 2). This effect is of course confounded with age, disease presentation, and progression.

Yet generally, direct referral is associated with a substantially shorter delay even if the referral diagnosis is wrong or unclear. Thus it seems worth considering a strategic shift by complementing awareness‐raising campaigns for involved medical disciplines (with only limited success in the past^14^) with actions facilitating early referral to expert centers.

As rare diseases generally share the challenge of diagnostic delay, the now increasingly established centers for rare diseases, which offer interdisciplinary expertise for many rare diseases under one umbrella, could become key players in this approach.

Our exploratory study is limited by its small sample size and the retrospective survey methodology. Yet, the participation of small, medium size, and large centers from three countries, the use of information retrieved independently from patients/parents and experts and the overall consistency of key data with larger studies indicate sufficient internal and external validity.

## 5 CONCLUSION

To our knowledge, this is the first systematic investigation on the diagnostic odyssey in IOPD and LOPD, which assesses the patients' as well as the expert's perspective. In conclusion, our study not only corroborates the substantial diagnostic delay in Pompe disease from previous publications,^14^, ^15^, ^16^, ^17^ but it gives clues toward an improved strategy to facilitate early diagnosis of Pompe disease, which aims for early referral of patients with unspecific symptoms to expert centers.

## CONFLICT OF INTERESTS

The authors of this manuscript declare no conflict of interests but disclose the following:

FBL has received honoraria and travel reimbursement from Actelion, BioMarin, Merck Serono, Sanofi Genzymem and Shire. AM has no conflict of interest. MR has received speaker honoraria from Genzyme and Shire. JH has received travel expenses from Shire, Sanofi/Genzyme, and Biomarin as well as honoraria from Shire. Her institute has received research/education funding from Shire. EM has received honoraria and/or consulting fees from Actelion, Alexion, BioMarin, Orphazyme, Sanofi Genzyme, and Shire. SG received travel expenses for presentations given at medical conferences, by Genzyme/Sanofi, Alexion, and Shire. TH received speakers' honoraria and travel reimbursement from Genzyme Switzerland. KR received honoraria as a consultant and an unrestricted research grant (not related to the present work) by Genzyme/Sanofi. He also received honoraria and travel expenses for presentations given at medical conferences by Genzyme/Sanofi and Shire. NK has received consulting fees from BioMarin, Sanofi Genzyme, and Shire. MH has received speaker honoraria from Genzyme, SOBI, and Shire and an unrestricted research grant (not related to the present work) from Nutricia Metabolics.

## INFORMED CONSENT

All procedures followed were in accordance with the ethical standards of the responsible committee on human experimentation (institutional and national) and with the Helsinki Declaration of 1975, as revised in 2000 (5).

## ANIMAL RIGHTS

This article does not contain any studies with animals performed by any of the authors.

## AUTHOR CONTRIBUTIONS

M.H., F.B.L., A.M. developed the concept and design, acquired data, performed analysis and interpretation of data, and wrote and finalized the manuscript. M.R., J.H., E.M., S.G., T.H., K.R., and N.K. acquired and interpreted the data, critically revised and approved the manuscript.
